# Bayesian deep learning for error estimation in the analysis of anomalous diffusion

**DOI:** 10.1038/s41467-022-34305-6

**Published:** 2022-11-07

**Authors:** Henrik Seckler, Ralf Metzler

**Affiliations:** 1 Institute for Physics & Astronomy, University of Potsdam, 14476 Potsdam-Golm, Germany; 2 Asia Pacific Centre for Theoretical Physics, Pohang, 37673 Republic of Korea

**Keywords:** Applied mathematics, Statistical physics

## Abstract

Modern single-particle-tracking techniques produce extensive time-series of diffusive motion in a wide variety of systems, from single-molecule motion in living-cells to movement ecology. The quest is to decipher the physical mechanisms encoded in the data and thus to better understand the probed systems. We here augment recently proposed machine-learning techniques for decoding anomalous-diffusion data to include an uncertainty estimate in addition to the predicted output. To avoid the Black-Box-Problem a Bayesian-Deep-Learning technique named Stochastic-Weight-Averaging-Gaussian is used to train models for both the classification of the diffusion model and the regression of the anomalous diffusion exponent of single-particle-trajectories. Evaluating their performance, we find that these models can achieve a well-calibrated error estimate while maintaining high prediction accuracies. In the analysis of the output uncertainty predictions we relate these to properties of the underlying diffusion models, thus providing insights into the learning process of the machine and the relevance of the output.

## Abstract (web summary)

Diffusive motions in complex environments such as living biological cells or soft matter systems can be analyzed with single-particle-tracking approaches, where accuracy of output may vary. The authors involve a machine-learning technique for decoding anomalous-diffusion data and provide an uncertainty estimate together with predicted output.

## Introduction

In 1905 Karl Pearson introduced the concept of the random walk as a path of successive random steps^1^. The model has since been used to describe random motion in many scientific fields, including ecology^2,3^, psychology^4^, physics^5^, chemistry^6^, biology^7^ and economics^8,9^. As long as the increments (steps) of such a random walk are independent and identically distributed with a finite variance, it will, under the *Central Limit Theorem* (CLT)^10^, lead to normal diffusion in the limit of many steps. The prime example of this is *Brownian motion*, which describes the random motion of small particles suspended in liquids or gases^11–14^. Amongst others, such normal diffusion entails that the *mean squared displacement* (MSD) grows linearly in time^15–17^, 〈**r**^2^(*t*)〉 ∝ *K*_1_*t*.

**Fig. 1:**
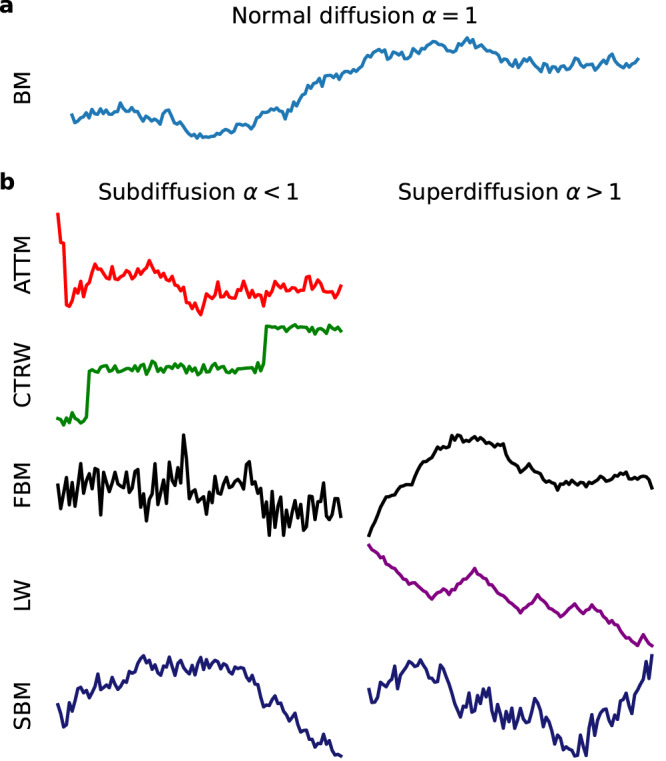
Illustration of different diffusion models. We show sample trajectories of normal (**a**) and anomalous (**b**) diffusion. All shown trajectories are corrupted by white Gaussian noise.

In practice however many systems instead exhibit a power law behaviour 〈**r**^2^(*t*)〉 ∝ *K*_*α*_*t*^*α*^ of the MSD^18–33^, indicating that one or several conditions of the CLT are not fulfilled. We refer to such systems as *anomalous diffusion*. A motion with anomalous diffusion exponent 0 < *α* < 1 is called subdiffusive, whereas for *α* > 1 it is referred to as superdiffusive (including ballistic motion with *α* = 2). In order to describe such systems mathematically, many models have been proposed, in which one or multiple conditions of the CLT are broken^24,25,34^. Some important examples (see the “Anomalous diffusion models” section for details) of such models are continuous-time random walk (CTRW)^35–37^, fractional Brownian motion (FBM)^38^, Lévy walk (LW)^39–42^, scaled Brownian motion (SBM)^43,44^ and annealed transient time motion (ATTM)^45^. Sample trajectories for these are shown in Fig. 1.

As each of these models correspond to different sources of anomalous diffusion, determining the model underlying given data can yield useful insights into the physical properties of a system^18–22,46,47^. Additionally one may wish to determine the parameters attributed to these models, the most sought-after being the anomalous diffusion exponent *α* and the generalised diffusion coefficient *K*_*α*_^18,48^. The used experimental data typically consist of single particle trajectories, such as the diffusion of a molecule inside a cell^7,30–33,47,49^, the path of an animal^2,3,50^ or the movement of stock prices^8,51^.

Plenty of techniques have been developed to tackle these tasks, usually through the use of statistical observables. Some examples include the ensemble-averaged or time-averaged MSD to determine the anomalous diffusion exponent and/or differentiate between a non-ergodic and ergodic model^52^, the p-variation test^53^, the velocity auto correlation for differentiation between CTRW and FBM^28^, the single trajectory power spectral density to determine the anomalous diffusion exponent and differentiate between models^54,55^, the first passage statistics^56^ and the codifference^57^. Such techniques may struggle when the amount of data is sparse and, with its rise in popularity, successful new methods using machine learning have emerged in recent years^58–60^.

**Fig. 2:**
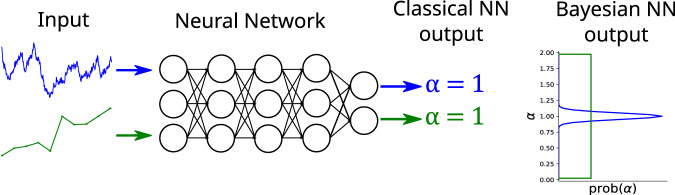
Illustration of the problem of reliability in deep learning. The illustration depicts the use of a neural network to predict the anomalous exponent *α* for two sample trajectories. Despite receiving severely different inputs, a classical neural network may still predict the same output (anomalous diffusion exponent *α* = 1) for both cases. The difference between the outputs only becomes clear when predicting not just the output itself but a distribution over all possible outputs, as it is done, for example, in Bayesian Deep Learning.

In an effort to generalise and compare the different approaches the *Anomalous Diffusion (AnDi) Challenge* was held in 2020^61,62^. The challenge consisted of three tasks, among them the determination of the anomalous diffusion exponent *α* and the underlying diffusion model from single particle trajectories. The entries included a wide variety of methods ranging from mathematical analysis of trajectory features^63,64^, to Bayesian Inference^65–67^, to a wide variety of machine learning techniques^59,68–77^. While the best results were achieved by deep learning (neural networks), this approach suffers from the so-called *Black Box Problem*, delivering answers without providing explanations as to how these are obtained or how reliable they are^78^. In particular, outputs are generated even in situations when the neural network was not trained for the specific type of motion displayed by the system under investigation. In this work, we aim at alleviating this problem by expanding the deep learning solutions to include an estimate of uncertainty in the given answer, as illustrated in Fig. 2. This is a feature that other techniques like *Bayesian Inference* can intrinsically provide^65–67^.

Such a reliability estimation is a well-known problem in machine learning. For neural networks the solutions vary from the calibration of neural network classifiers^79–82^, to using an ensemble of neural networks and obtaining an uncertainty from the prediction spread^83^, to fully modelling the probability distribution of the outputs in *Bayesian Neural Networks*^84^. In recent years the latter has been expanded to be applicable to deep neural networks without resulting in unattainable computational costs. These *Bayesian Deep Learning* (BDL) techniques approximate the probability distribution by various means, for instance, by using drop out^85,86^ or an ensemble of neural networks^83^. We here decided on using a method by Maddox et al. named *Stochastic Weight Averaging Gaussian* (SWAG), in which the probability distribution over the network weights is approximated by a Gaussian, obtained by interpreting a stochastic gradient descent as an approximate *Bayesian Inference* scheme^87,88^. We find that these methods are able to produce well-calibrated uncertainty estimates, while maintaining the prediction performance of the best *AnDi-Challenge* solutions. We show that analysing these uncertainty estimates and relating them to properties of the diffusion models can provide interesting insights into the learning process of the machine.

The paper is structured as follows. A detailed analysis of our results for regression and classification is presented in the “Results” section. These results are then discussed and put into perspective in the “Discussion” section. A detailed explanation of the utilised methods is provided in the “Methods” section. Here we provide a brief introduction to the different anomalous diffusion models in the subsection “Anomalous diffusion models” and the used SWAG method in the subsection “Uncertainties in deep learning”. Subsequently, the neural network architecture and training procedure used in our analysis is presented in the subsection “Neural network architecture and training”. The Supplementary Information details the reliability assessment methods and provides Supplementary Figures.

## Results

In the following, we employ the methods detailed in the “Methods” section to construct the *Multi-SWAG*^88^ models and use these to determine the anomalous diffusion exponent *α* or the diffusion model of computer-generated trajectories. We also provide detailed error estimates to qualify the given outputs. These estimates consist of a standard deviation for regression and model probabilities for classification. The trajectories are randomly generated from one of the five diffusion models: continuous-time random walk (CTRW)^35–37^, fractional Brownian motion (FBM)^38^, Lévy walk (LW)^39–42^, scaled Brownian motion (SBM)^43,44^ or annealed transient time motion (ATTM)^45^, as detailed in the “Anomalous diffusion models” section. We evaluate the performance of the uncertainty estimation for the regression of the anomalous diffusion exponent (see the “Regression” section) and the classification of the diffusion model (see the “Classification” section). We find that for both classification and regression the added error estimate does not diminish performance, such that we can still achieve results on par with the best *AnDi-Challenge* competitors. The added error estimate proves to be highly accurate even for short trajectories, an observation that merits a detailed investigation of its behaviour. We analyse the error prediction behaviour depending on the diffusion model, anomalous diffusion exponent, noise and trajectory length in order to obtain insights into the learning process of the machine. To differentiate between error predictions due to model uncertainties and those inherent in each model, we further analyse the predicted uncertainties for the inference of the anomalous diffusion exponent with known ground truth diffusion model in the “Single model regression” section. We show that the observed dependencies can be attributed to specific properties of the underlying diffusion models.

### Regression

**Fig. 3:**
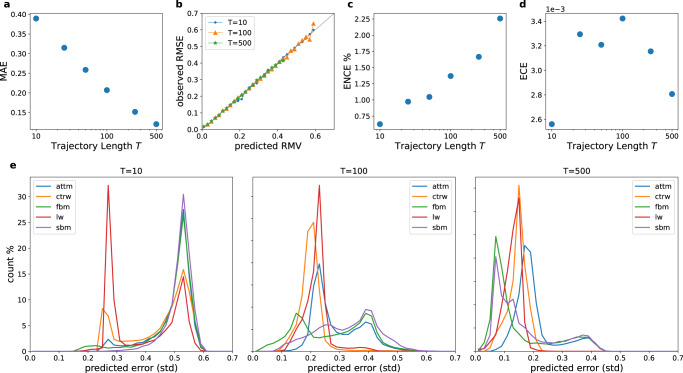
Performance evaluation for the regression of the anomalous exponent *α*. **a** Mean absolute error (MAE), **b** reliability diagram and expected (**c**) normalised and (**d**) non-normalised calibration error (ENCE/ECE)^82^ achieved by *Multi-SWAG* (see Supplementary Information for detailed definitions). Results are plotted for different trajectory lengths *T* by averaging over 10^5^ test trajectories each. The MAEs (**a**) show a decreasing trend with trajectory length, with results close to those achieved in the *AnDi-Challenge*, reaching an MAE of 0.14 for *T* = 500. To judge the error prediction performance, the reliability diagram (**b**) depicts the observed root mean squared error (RMSE) as a function of the predicted root mean variance (RMV), showing that even for very short trajectories *T* = 10 an error prediction close to the ideal (grey line) is achieved. The reliability diagram can be summarised in a single value using the ECE/ENCE. The ENCE/ECE characterises the mean difference between predicted and observed errors, either normalised to obtain a relative error (ENCE) or as an absolute (ECE). As visible in (**b**), we obtain good error predictions with an ENCE between 0.6 and 2.3% depending on the trajectory length. The increase in ENCE with trajectory lengths can be attributed to the decrease in MAE (and therefore predicted errors), while the unnormalised ECE only shows a slight trend of decreasing with trajectory length. The low ECE for *T* = 10 is due to the high number of trajectories predicted with near maximal error. **e** Predicted error histogram for inferring the anomalous diffusion exponent *α* when the underlying model is unknown. The figure shows the distribution of the error as predicted by *Multi-SWAG* trained on all models. Each subplot shows the results for a different trajectory length *T*, as obtained from predictions on 10^5^ trajectories.

In order to quantify the performance of our *Multi-SWAG*^88^ models we test them on a new set of computer-generated trajectories using the andi-datasets package. For the general prediction of the anomalous diffusion exponent *α* we obtain results comparable to the best participants in the *AnDi-Challenge*^59,62,63,65–77^. The achieved mean average error for different trajectory lengths in Fig. 3a shows an expected decreasing trend with trajectory length.

To analyse the performance of the error prediction we use a reliability diagram^79–81^ in Fig. 3b. The figure depicts the observed root mean squared error (RMSE) from the ground truth exponent as a function of the predicted root mean variance (RMV) (see Supplementary Information for detailed definitions). Grouping together predictions within a range of 0.02, we see results close to the ideal of coinciding predictions and observations. As is to be expected, longer trajectories show smaller predicted errors, yet, the higher errors for very short trajectories of only 10 time steps are still predicted remarkably well. The results of the reliability diagram can be summarised using the Expected Normalised Calibration Error (ENCE)^82^, which calculates the normalised mean deviation between observed and predicted uncertainty. Figure 3c shows a low ENCE between 0.6% and 2.3%, which increases with trajectory length. This increase can be attributed to the decrease in predicted standard deviations, which results in a higher normalised error due to the fact that the unnormalised expected calibration error (ECE) only shows a slight decrease with trajectory length, as can be seen in Fig. 3d.

In order to better understand how the network obtained these predictions, it proves useful to observe the frequency of predicted standard deviations in Fig. 3e. The histograms there show how often which error is predicted for different ground truth models.

For very short trajectories (*T* = 10) we observe a split of the predictions into two peaks. This observation can be attributed to the different priors of the ground truth models. If the network can confidently identify the trajectory as belonging to one of the only sub-/superdiffusive models (CTRW/LW/ATTM), it can predict (and achieve) a smaller error due to the reduced range of possible *α*-values. From the different heights of this second peak, we can also conclude that, for very short trajectories, LW is easier to identify than CTRW or ATTM. This is likely due to the fact that LWs have long structures without a change in direction, that can be fairly easily identified, while CTRWs with long resting times will be particularly camouflaged by the noise and ATTMs without jumps in the diffusivity will be indistinguishable from normal diffusion. Other than identifying the model the network does not seem to gain much information from these short trajectories as the two peaks are close to the maximum predicted errors one would expect with respect to the priors. FBM trajectories, however, are an exception to this, as one may already see a small amount of very low predicted errors, which will be further studied in the “Single model regression” section.

When increasing the trajectory lengths we see lower error predictions for all models. Both FBM and SBM achieve lower predicted errors than the other three models, despite the larger range of *α*, which may be attributed to the fact that they do not rely on hidden waiting times, in contrast to the other three models. While we see FBM’s accuracy increasing faster than SBM’s at the beginning for *T* = 100, we obtain similar predicted errors for the two models for *T* = 500. This may be caused by SBM being highly influenced by noise (see “Single model regression”) and thus easier to be confused with ATTM, since both feature a time-dependent diffusivity. The errors introduced by model confusion can also be observed in the persisting second peak. As we will see below, this peak can be understood as a property of ATTM. An ATTM trajectory with no jumps in diffusivity, which will occur more often for very subdiffusive trajectories (small *α*), will be indistinguishable from normal diffusion with *α* = 1, thereby introducing a large error. Due to the uncertainty in the underlying model this predicted error is also present for both FBM and SBM, both exhibiting ordinary Brownian Motion for *α* = 1.

Analogously to the other models the predicted error for LW and CTRW reduces with increased trajectory length. CTRW shows less error than LW for *T* = 100, which may be attributed to the smaller prior used for the CTRW trajectories 0.05 ≤ *α* ≤ 1 compared to LW 1 < *α* ≤ 2. For *T* = 500 this difference vanishes, as the importance of different priors decreases with better accuracy, and we even see a slightly lower predicted error for LW.

#### Single model regression

**Fig. 4:**
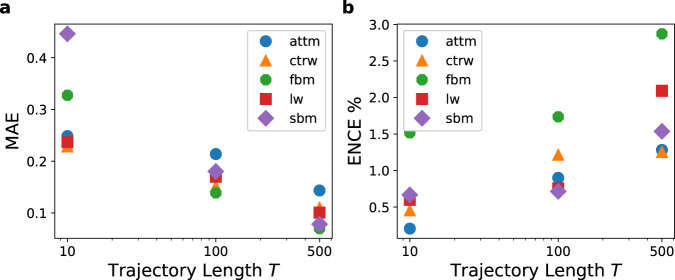
Performance evaluation for regression when the underlying model is known. **a** Mean absolute error (MAE) and **b** expected normalised calibration error (ENCE)^82^ achieved by the *Multi-SWAG* models trained on only one model, plotted for different trajectory lengths *T* by averaging over 5 × 10^4^ (FBM, SBM) or 4 × 10^4^ (ATTM, LW, CTRW) test trajectories each. The ENCE characterises the mean difference between predicted and observed errors. As was the case for the unknown ground truth model (Fig. 3), we can achieve a small calibration error below 3%. The MAE shows the expected results with regards to the histograms in Fig. 5.

**Fig. 5:**
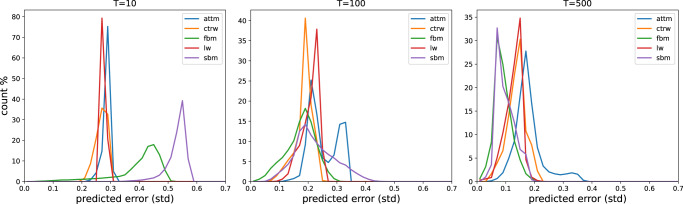
Predicted error histogram for inferring the anomalous diffusion exponent when the underlying model is known. The figure shows the distribution of the error as predicted by neural networks trained individually for each model. The histograms are obtained from predictions on 5 × 10^4^ (FBM/SBM) or 4 × 10^4^ (ATTM/LW/CTRW) trajectories.

**Fig. 6:**
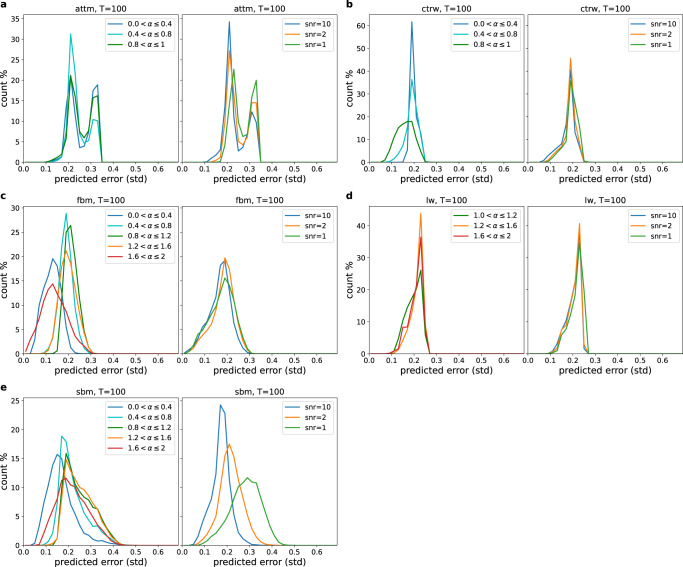
Predicted error histogram for known ground truth model, split by exponent and noise for trajectories of length *T* = 100. Each of the figures [**a** FBM, **b** SBM, **c** ATTM, **d** CTRW, **e** LW] shows the results for one of the five different ground truth models, obtained from predictions on 5 × 10^4^ (FBM/SBM) or 4 × 10^4^ (ATTM/LW/CTRW) trajectories.

In order to differentiate between errors originating from the model uncertainty and errors specific to an individual model, it proves useful to perform a regression of the anomalous diffusion exponent *α* on only a single diffusion model with networks trained on only that model. As before we are able to obtain small ENCEs below 3%, as seen in Fig. 4. Due to this low calibration error the achieved MAEs in Fig. 4 largely resemble the predicted errors in the histograms in Fig. 5, which will be discussed in detail in the following. In addition, we analyse the change in predicted errors with respect to the ground truth exponent and the noise, using the histograms in Fig. 6a–e for trajectories of length *T* = 100, as well as Supplementary Fig. S1 for lengths *T* = 10 and 500.

##### FBM

As one expects, due to the larger prior, FBM’s error predictions for very short trajectories (*T* = 10) are larger than the three exclusively sub- or superdiffusive models. Compared to SBM and the performances for unknown ground truth models in Fig. 3e, these errors are, however, remarkably low, showing that, while the correlations for very short trajectories were not noticeable enough to identify them as FBM above, they are enough to significantly improve the performance when they are known to be FBM trajectories. Additionally one may also notice a small percentage of trajectories assigned with very low predicted error, which can also be seen for longer trajectories but is less noticeable. As before, we see that the predictions quickly improve for longer trajectories and ultimately reach better results than for ATTM, LW, or CTRW.

By studying the dependence of the predicted error on the ground truth exponent in Figs. 6a and S1, we can attribute the low error predictions to the very super-/subdiffusive trajectories, for which correlations are apparent. This feature occurs despite of the fact that for short trajectories only the superdiffusive trajectories contribute, which is likely caused due to anticorrelations in short trajectories being similar to noisy trajectories. Concerning the dependence on noise we only see a slight increase in the predicted accuracy for lower noise regardless of the trajectory length, although the possibility of high noise likely influences the predictions, as explained above.

##### SBM

Similar to FBM, due to the large prior, SBM trajectories start with high error predictions for very short trajectories in Fig. 5. In contrast to FBM, however, these predictions are much higher, since a change in diffusivity will be hard to detect for few time steps. When increasing the lengths, the predictions improve, getting close to those for FBM for *T* = 500. Similar to above, we also observe a noticeably broad distribution of errors, this time however to the right side of the peak. We can explain this broadness by examining the noise dependence of the predictions in Fig. 6b (and S1). We see a large difference between predicted errors depending on noise. For example, for length *T* = 100 we obtain a mean predicted standard deviation of ≈0.032 for low noise (snr = 10) and ≈0.082 for high noise (snr = 1), more than doubling the error. We can attribute this effect to the influence of static noise on a trajectory, whose increments increase/decrease over time for super-/subdiffusive trajectories. This will effectively hide part of the data under high noise, reducing the number of effectively useful data points.

When observing the dependence of the predicted error on the ground truth exponent in Fig. 6b we can see better predictions for the more pronouncedly sub- and superdiffusive cases for length *T* = 100, showing that despite the fact that part of these trajectories are hidden under the noise, the large increase/decrease in diffusivity still makes these trajectories easier to identify. One should also keep in mind that while these will be very noisy at one end, they will also be less noisy at the other end. The network does, however, assign a lower predicted error for subdiffusive trajectories than for superdiffusive ones, for which the difference increases for larger snr. This may indicate, that the subdiffusive decrease in diffusivity (∝ 1/*t*^1−*α*^ → 1/*t* for *α* → 0) is easier to identify than the superdiffusive increase (∝ *t*^*α*−1^ → *t* for *α* → 2). The former will have a larger portion of the trajectory hidden under the noise with a steep visible decrease at the beginning, while the latter will increase more slowly, leading to a smaller hidden portion but also making the non-hidden part less distinct and the transition between more ambiguous.

##### ATTM

In Fig. 5 we see a behaviour for ATTM similar to what was discussed in the previous section. This time the histogram starts for short trajectories as a single peak close to the maximum prediction possible with respect to the prior. With increasing length the peak splits into two peaks, where the second peak, as discussed above, originates from subdiffusive ATTM trajectories with few or no jumps in the diffusivity. This second peak decreases in volume for very long trajectories, since observing no jumps becomes rarer and it becomes easier to identify the still occurring, albeit small, jumps in normal-diffusive (*α* = 1) ATTM trajectories. The second point should also be the reason why the right peak is less pronounced than in the case of unknown underlying model in Fig. 3e, as it is easier to confuse subdiffusive ATTM with normal-diffusive FBM/SBM than with normal-diffusive ATTM.

For the *α*-dependence in Figs. 6c and S1 we can see that, as expected, the right peak is more pronounced for sub- and normal-diffusive trajectories. For length *T* = 500 (Fig. S1) we also see that the lowest errors originate from close to normal-diffusive trajectories, as these will exhibit more jumps and thereby allow to identify more waiting times. As for the influence of the noise, in Fig. 6c (S1) we see a slight increase of the uncertainty with higher noise, as well as the right peak being more pronounced for higher noise, likely due to the fact that the noise obscures the smaller jumps occurring in normal-diffusive ATTM.

##### CTRW

As seen in Fig. 5 CTRW shows a single peak, whose location shifts to lower predicted errors with increasing trajectory length. When examining the dependence on the ground truth *α* value and noise in Figs. 6d and S1, one can see that an increase in the noise will have little effect on the predictions, only leading to a slight increase in the predicted error. The largest difference is observed for very short trajectories in Fig. S1, likely for the fact that the low noise here allows one to detect the very few jumps in the short trajectories. The exponent *α*, however, has a higher influence on the error predictions. One can observe that the predicted error will be smaller for exponents closer to normal diffusion, arguably as more jumps occur in this case.

##### LW

The LW evaluation in Fig. 5 exhibits similar behaviour to the CTRW, showing a single peak shifting toward lower predicted errors. As discussed above the predictions for LW are slightly worse than for CTRW in the beginning, which we attribute to the difference in the prior. In Figs. 6e and S1, we see little to no influence of the noise on the error predictions. From these figures one may also obtain a similar, though much less pronounced, behaviour in dependence of the ground truth *α* as for CTRW. As was the case there we see lower predictions for exponents close to normal diffusion, as more hidden waiting times can be observed. Interestingly in Fig. S1 we see that for long trajectories the predicted error will also be reduced for very superdiffusive trajectories. In part, this can be attributed to the distinct ballistic *α* = 2 LW, but should also be caused by the noise as superdiffusive LW with a few very long jumps is, in contrast to CTRW with few jumps, not highly influenced by noise.

### Classification

**Fig. 7:**
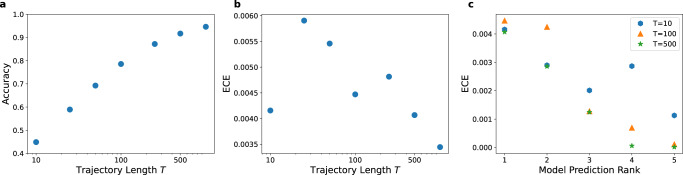
Performance evaluation for the classification of the diffusion model. **a** Total accuracy and **b** expected calibration error (ECE)^80,81^ (see Supplementary Information for detailed definitions) achieved by *Multi-SWAG*, plotted for different trajectory lengths *T* by averaging over 10^5^ trajectories each. The ECE describes the difference one may expect between the predicted confidence and the observed accuracy. As before we achieve a low calibration error between 0.3% and 0.6%. The classification accuracy improves the longer the trajectory, achieving results similar to the best scoring models in the *AnDi-Challenge*^62^. **c** Expected calibration error (ECE)^80,81^ achieved for lower-ranked predictions, meaning those models that were not assigned the highest confidence. A prediction of rank *i* corresponds to the output with the *i*th highest confidence. Even these predictions show low calibration errors below 0.5%. The vanishing ECE for the 4th and lower-ranked predictions of long trajectories are caused by them being correctly assigned a 0% probability.

Complementing the discussion of the regression in section “Regression”, we now evaluate the trained *Multi-SWAG* models for classification on the test data set. The achieved accuracies depicted in Fig. 7a are in line with the best-performing participants of the *AnDi-Challenge*^59,62,63,65–77^. As one would expect the achieved accuracy increases with trajectory length, starting from 44.9% for *T* = 10 and reaching 91.7% for *T* = 500. In Fig. 7b, we also see a very good performance for error prediction, the expected calibration error only ranging from 0.3 to 0.6 percentage points. The ECE generally shows a decreasing trend with increasing trajectory length, although very short trajectories of *T* = 10 also achieved a low ECE, likely due to a high number of trajectories predicted with very low confidences. Remarkably even the confidences of the lower-ranked predictions, relating to those models that were not assigned the highest confidence, achieve similarly low ECEs in Fig. 7c.

**Fig. 8:**
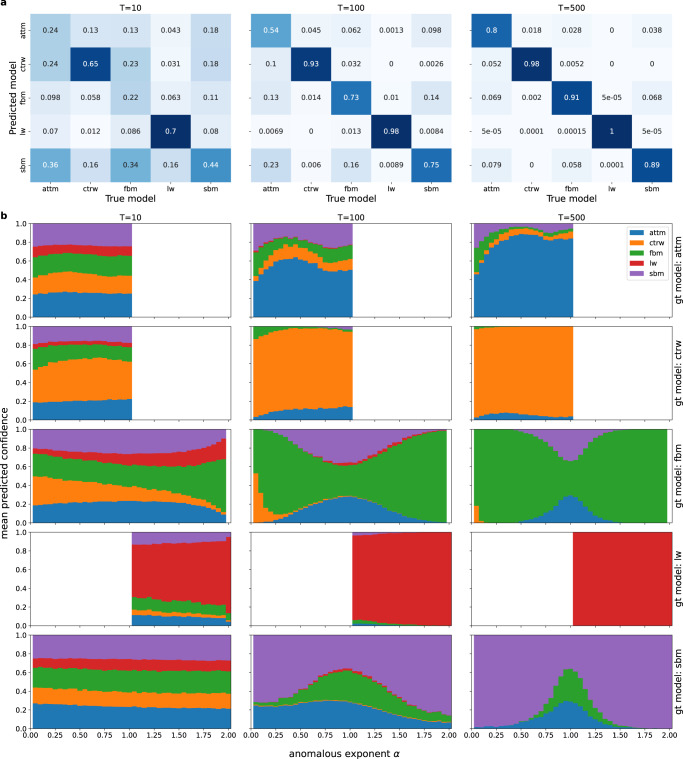
Analysis of the classification behaviour. **a** Confusion matrices for different trajectory lengths. The entries indicate the relative frequency of model predictions (row) given a ground truth model (column). The matrices are obtained from *Multi-SWAG* predictions on a total of 10^5^ test trajectories for each length. **b** Mean confidences for different ground truth (gt) models in dependence of the ground truth anomalous diffusion exponent *α*. From the figure one can infer the confidence for the different models shown as coloured bars as assigned by *Multi-SWAG*. The illustrations are plotted for different trajectory lengths by averaging over a total of 10^5^ trajectories for each length, translating to 2 × 10^4^ trajectories for each ground truth model and length.

To further analyse the performance and error prediction, we show the confusion matrices in Fig. 8a and the mean predicted confidences in Fig. 8b. The confusion matrices depict how often a model is predicted given a specific ground truth models, thereby showing how often and with which model each model is confused. As such matrices do not consider the predicted confidences and have already been thoroughly examined in other works^59,62,63,65–77^, we will focus our investigation on the second Fig. 8, which illustrates the mean predicted confidences of each model for different ground truth models in dependence of the true anomalous diffusion exponent *α*. Note that while the mean confidence will in part reflect the predictions in the confusion matrix, this quantity also provides additional, complementary information, as the confusion matrix only considers the models with the highest membership score. In the following we analyse the results for different ground truth models.

#### ATTM

ATTM trajectories generally show the worst classification performance of the range of models studied here. For very short trajectories (*T* = 10) we see that the mean confidence splits among all models with the lowest probabilities being assigned to the exclusively superdiffusive LW. Reflecting the confusion matrix, the confidences for SBM are the highest, likely due to both SBM and ATTM featuring a time-dependent diffusivity. For longer trajectories, we see the confidence for FBM and SBM rise for lower *α*, which, as explained above, can be attributed to that fact that ATTM without jumps is indiscernible from ordinary Brownian motion. The confusions for CTRW, which are most present for moderately subdiffusive to normal-diffusive trajectories, can be attributed to the fact that both models feature hidden waiting times and that short periods of high diffusivity in ATTM appear similar to jumps in CTRW.

#### CTRW

Reflecting the high accuracies in the confusion matrices, we observe high confidence for CTRW for longer trajectories (*T* ≥ 100). For very subdiffusive trajectories we see an increase in the predicted probability for FBM, which can be explained by the fact that CTRWs without jumps solely consist of noise, which corresponds to an FBM trajectory with *α* = 0. We can also observe a similar confusion behaviour between ATTM and CTRW as was described for ATTM. For very short trajectories (*T* = 10) the confidences for CTRW are relatively high as compared to the other ground truth models, and they increase with higher anomalous diffusion exponent, which we attribute to the increase in jump frequency with higher *α*. Here confidences for models other than CTRW are split between ATTM, FBM and SBM with only small confidences assigned to the solely superdiffusive LW.

#### FBM

Similarly to what we described in the “Regression” section, for shorter trajectories, we see a large difference in FBM confidences for very sub- and superdiffusive *α*. We there hypothesised this difference to be caused by the inability to discern very subdiffusive trajectories from noise. This can be confirmed here, as subdiffusive trajectories show the highest confusion with CTRW, which without jumps solely consists of noise. For very short trajectories we see an increase in LW confidence with increasing *α*, likely due to highly correlated, very short FBM trajectories looking similar to LW trajectories without jumps. For longer trajectories one can observe low FBM confidence at and around *α* = 1, which is caused by FBM’s convergence to normal diffusion and leads to split uncertainties between FBM, SBM and ATTM. One should note that the ATTM confidences here would not correspond to a normal-diffusive ATTM but rather to a strongly subdiffusive ATTM without jumps in diffusivity, as is evidenced by the mean confidences for ATTM ground truth trajectories.

#### LW

In accordance to the high accuracies observed in the confusion matrices, the mean confidences for LW are high even for relatively short trajectories. These high confidences occur, as LW is easily identifiable even with few jumps. In fact the increase in confidence with rising anomalous diffusion exponent suggests that LW trajectories are easier to identify when fewer jumps occur, which is in contrast to ATTM/CTRW, which both feature a decrease in model confidence with fewer jumps. One should also note the jump in confidence caused by ballistic LW (*α* = 2).

#### SBM

As was the case for FBM, for longer SBM trajectories we see the same confusion pattern between SBM, ATTM and FBM at and around normal diffusion *α* = 1. However, we also see relatively high assigned confidences for ATTM for subdiffusive trajectories, which we again attribute to both models featuring time-dependent diffusivities. We see low confidence for SBM for very short trajectories, likely due to a change in diffusivity not being noticeable for so few data points.

In Supplementary Fig. S2a–c, we include error histograms similar to those used for regression. These resemble the already discussed behaviour and indicate in addition that the distribution of predicted errors often features a large number of trajectories predicted with high confidences of 95% to 100%.

## Discussion

The *AnDi-Challenge* demonstrated the power of a rich arsenal of successful machine learning approaches to analyse anomalous diffusion trajectories. These proposed models, however, all suffered from a lack of explainability due to the Black Box problem, providing answers without explanation, which also leads to an uncertainty in the reliability and usefulness of the approaches for real-world systems.

Here we expanded the successful machine learning solutions featuring in the *AnDi-Challenge* by adding a reliability estimate to the predictions of the machine. This estimate was obtained by modelling aleatoric and epistemic uncertainties in the model, the latter by using a Bayesian machine learning techniques called *Multi Stochastic Weight Averaging Gaussian*. We showed that the resulting model is able to provide accurate error estimates even for very uncertain predictions when tested on separate, but identically distributed, test data sets. It was also demonstrated that these uncertainty predictions provide an additional tool to understand how machine learning results are obtained. By analysing the prediction behaviour with respect to diffusion model, noise, anomalous diffusion exponent and trajectory length, we were able to relate its cause to the properties of the underlying anomalous diffusion models. This analysis also indicated that a network trained to predict the anomalous diffusion exponent will already learn to differentiate between the anomalous diffusion models. In our study, we also introduced the mean confidence diagrams and showed that they provide vital information complementary to confusion matrices.

For future works testing the *Multi-SWAG* models on diffusion data whose dynamics are not included in the training set will be an interesting field of study. Such data may include trajectories generated with different diffusion models, a subordination or superposition of models or with changing models. Results here will indicate, what behaviour one should expect when using these models on experimental data, as such data will rarely exactly follow the theoretical models. Naturally though this can and should not replace the need to test the developed methods here as well. Similarly, it might be of interest to analyse the results obtained when applying these methods to "poisoned” (faulty) test data, e.g., when non-Gaussian errors contaminate the data, non-trained stochastic mechanisms are included, or the analysed time series have missing points. As one would expect, this leads to a higher predicted error due to the epistemic uncertainty, as described in the “Uncertainties in deep learning” section. Quantifying such errors systematically will be an interesting question for the future. We also mention that applying the used BDL methods to the feature-based approaches for decoding anomalous diffusion data brought forth recently^60,75–77^ and analysing error prediction performance as well as the impact of the different features on these error predictions, could also provide interesting insights. Another interesting avenue could be provided by the third task of the *AnDi-Challenge*, which consisted of predicting the change point of a diffusion trajectory switching models and/or exponent. Recent studies suggest that sequence to sequence networks, predicting trajectory properties at each time step, are suited to solve this task^62^. Here BDL might provide an advantage in addition to the error estimate, as one would expect the predicted uncertainty to maximise at the change point and thereby simplify its determination.

## Methods

### Anomalous diffusion models

For comparability, the models considered in this work are the same as those in the *AnDi-Challenge*^61,62^. The trajectories are generated from one of the 5 models below, all producing an MSD of the form 〈**r**^2^(*t*)〉 ∝ *K*_*α*_*t*^*α*^. Examples for each model are shown in Fig. 1.

#### CTRW

The continuous-time random walk (CTRW) is defined as a random walk, in which the times between jumps and the spatial displacements are stochastic variables^35–37^. In our case, we are considering a CTRW for which the waiting time distribution Ψ(*τ*) features a power law tail Ψ(*τ*) ∝ *τ*^−1−*α*^ with scaling exponent 0 < *α* < 1, thereby leading to a diverging mean waiting time $\int\nolimits_{0}^{\infty }\tau {{\Psi }}(\tau )d\tau=\infty$. The spatial displacements follow a Gaussian law.

#### LW

The Lévy walk (LW) is a special case of a CTRW. As above we consider power law distributed waiting times Ψ(*τ*) ∝ *τ*^−1−*σ*^, but the displacements are correlated, such that the walker always moves with constant speed *v* in one direction for one waiting time, randomly choosing a new direction after each waiting time. One can show that this leads to an anomalous diffusion exponent *α* given by^42^

1 $$\alpha=\left\{\begin{array}{ll}2&{{{{{{{\rm{if}}}}}}}}\,0\; < \;\sigma\; < \;1\,({{{{{{{\rm{ballistic}}}}}}}}\,{{{{{{{\rm{diffusion}}}}}}}})\\ 3-\sigma &{{{{{{{\rm{if}}}}}}}}\,1\; < \;\sigma\; < \;2\,({{{{{{{\rm{superdiffusion}}}}}}}}).\end{array}\right.$$

#### FBM

Fractional Brownian motion (FBM) is characterised by a long-range correlation between the increments. It is created by using fractional Gaussian noise for the increments given by

2 $$\langle {\xi }_{fGn}(t){\xi }_{fGn}(t+\tau )\rangle \sim \alpha (\alpha -1){K}_{\alpha }{\tau }^{\alpha -2}$$

for sufficiently large *τ*, where *α* is the anomalous diffusion exponent and *K*_*α*_ is the generalised diffusion constant^38^.

#### SBM

Scaled Brownian motion (SBM) features the time-dependent diffusivity *K*(*t*) = *αK*_*α*_*t*^*α*−1^, equivalent to the Langevin equation

3 $$\frac{dx(t)}{dt}=\sqrt{2K(t)}\xi (t),$$

where *ξ*(*t*) is white, zero-mean Gaussian noise^44^.

#### ATTM

Similar to SBM, the annealed transient time motion (ATTM) features a diffusion coefficient *D* varying over time. But in contrast to SBM, the change in diffusivity is random in magnitude and occurs instantaneously in a manner similar to the jumps in a CTRW. Here we consider diffusion coefficients sampled from the distribution *P*(*D*) ∝ *D*^*σ*−1^ and use a delta distribution of waiting times *P*(*τ*) ∝ *δ*(*τ* − *D*^−*γ*^), with *σ* < *γ* < *σ* + 1. As shown in ref. ^45^, this leads to subdiffusion with *α* = *σ*/*γ*.

We use the andi-datasets Python package for the implementation of these models^89^. In an effort to simulate conditions closer to experimental situations, all data are corrupted by white Gaussian noise with the signal-to-noise strength ratio snr ∈ {1, 2, 10}. Given the trajectory *x*_*t*_, we obtain the noisy trajectory $\tilde{x}(t)=x(t)+\xi (t)$ with the superimposed noise

4 $$\xi (t) \sim \frac{{\sigma }_{{{\Delta }}x}}{{{{{{{{\rm{snr}}}}}}}}}{{{{{{{\mathcal{N}}}}}}}}(0,1){{{{{{{\rm{,}}}}}}}}$$

where *σ*_Δ*x*_ is the standard deviation of the increment process Δ*x*(*t*) = *x*(*t* + 1) − *x*(*t*). We consider trajectories generated with anomalous diffusion exponents *α* ∈ {0.05, 0.10, . . . , 1.95, 2}. Note however that only SBM is applied to the whole range of *α* values. CTRW and ATTM are only sub- or normal-diffusive (*α* ≤ 1), LW is superdiffusive (*α* > 1) and ballistic (*α* = 2) FBM is not considered here. This entails that data sets with a mixture of models cannot be equally distributed with respect to the anomalous diffusion exponents and underlying models at the same time. In this work, we choose the prior distributions of models and exponents such that they conform with those used in the *AnDi-Challenge*, where the priors were chosen to simulate no prior-knowledge for the given task. This entails that the data set used for the classification tasks is equally distributed with respect to models but not among anomalous diffusion exponents, and vice versa for the data set used for the regression of *α*. Subdiffusive trajectories are therefore overrepresented in the classification data sets, while FBM and SBM will be overrepresented for regression.

### Uncertainties in deep learning

In short, a neural network in deep learning is a function approximator, where the output *f*_*θ*_(*x*_*i*_) of the neural network given inputs *x*_*i*_ is optimised to minimise some loss function ${{{{{{{\mathcal{L}}}}}}}}$. This is achieved by fitting the function parameters (weights) *θ* of the neural network, usually by utilising the stochastic gradient descent algorithm or a variation of it^90^.

In Bayesian Deep Learning, one differentiates between two major types of uncertainty named aleatoric and epistemic uncertainty^91,92^.

#### Aleatoric uncertainty

Aleatoric uncertainty refers to the uncertainty inherent in the system underlying the data, caused, for example, by noise or an inherent stochasticity of the system. This kind of uncertainty needs to be included in the output of the neural network model. We then minimise the negative log-likelihood loss

5 $${{{{{{{{\mathcal{L}}}}}}}}}_{{{{{{{{\rm{nll}}}}}}}}}=-\mathop{\sum}\limits_{i}\log p(\,{\hat{y}}_{i}|\,{f}_{\theta }({x}_{i})),$$

where ${\hat{y}}_{i}$ is the target output and *f*_*θ*_(*x*_*i*_) is the prediction of the neural network given input *x*_*i*_ and weights *θ*^93^.

For regression problems, the commonly used models output only a predicted value and optimise the network to minimise either the mean absolute error or the mean squared error^94^. In order to model aleatoric uncertainty we modify the network to output mean and variance of a Gaussian predictive distribution, instead of just predicting a single value (while a Gaussian distribution will often not be a precise approximation, it suffices to obtain well-calibrated estimates for the standard deviation). When $p(\,{\hat{y}}_{i}|\,{f}_{\theta }({x}_{i})) \sim {{{{{{{{\mathcal{N}}}}}}}}}_{{\mu }_{i},{\sigma }_{i}}(\,{\hat{y}}_{i})$, we minimise the negative log-likelihood, which becomes the Gaussian negative log-likelihood loss

6 $${{{{{{{{\mathcal{L}}}}}}}}}_{{{{{{{{\rm{gnll}}}}}}}}}=\mathop{\sum}\limits_{i}\frac{1}{2}\left(\log ({\sigma }_{i}^{2})+\frac{||{\mu }_{i}-{\hat{y}}_{i}|{|}^{2}}{{\sigma }_{i}^{2}}\right)+{{{{{{{\rm{const}}}}}}}},$$

where *μ*_*i*_ and *σ*_*i*_ are the mean and variance outputs of the neural network for input *x*_*i*_^95^.

The commonly used models for classification already output an aleatoric error. We train the model to output membership scores for each class in a so-called *logit* vector *z*_*i*_ = *f*_*θ*_(*x*_*i*_), from which the class probabilities can be obtained via a normalised exponential (*softmax*) function

7 $${p}_{i,k}=\frac{\exp {z}_{i,k}}{{\sum }_{k}\exp {z}_{i,k}},$$

where _*pi*,*k*_ is the predicted probability of class *k* given input *x*_*i*_. From the negative log-likelihood loss we then obtain the *cross entropy loss*

8 $${{{{{{{{\mathcal{L}}}}}}}}}_{{{\rm{cel}}}}=-\mathop{\sum}\limits_{i,k}{\hat{y}}_{i,k}\log ({p}_{i,k}),$$

where ${\hat{y}}_{i,k}$ is a binary indicator ${\hat{y}}_{i,k}={\delta }_{{j}_{i}k}$ of the true class *j*_*i*_ of input *x*_*i*_.

#### Epistemic uncertainty and stochastic weight averaging Gaussian (SWAG)

Epistemic uncertainty refers to the uncertainty caused by an imperfect model, for example due to a difference between training and test data or insufficient training data. In Bayesian Deep Learning we model this error by assigning an uncertainty to the inferred neural network weights. If $p(\theta|{{{{{{{\mathcal{D}}}}}}}})$ is the probability distribution over the weights *θ* given data ${{{{{{{\mathcal{D}}}}}}}}$, we obtain

9 $$p(\,y|{x}_{i},{{{{{{{\mathcal{D}}}}}}}})=\int d\theta p(\,y|{x}_{i},\theta )p(\theta|{{{{{{{\mathcal{D}}}}}}}}).$$

In practice this integral is approximated by *Monte Carlo (MC) integration*^96^

10 $$p(\,y|{x}_{i},{{{{{{{\mathcal{D}}}}}}}})\;\approx\, \frac{1}{M}\mathop{\sum }\limits_{m=1}^{M}p(\,y|{x}_{i},{\theta }_{m}),$$

where the weights *θ*_*m*_ are sampled from the posterior $p(\theta|{{{{{{{\mathcal{D}}}}}}}})$ and *M* is the number of MC-samples. Mathematically this posterior is given by *Bayes’ rule*^97^

11 $$p(\theta|{{{{{{{\mathcal{D}}}}}}}})=\frac{p({{{{{{{\mathcal{D}}}}}}}}|\theta )p(\theta )}{p({{{{{{{\mathcal{D}}}}}}}})}.$$

However, as calculating the posterior becomes intractable for large networks and data sets, we need to approximate it. For this purpose Maddox et al. proposed a method named *Stochastic Weight Averaging Gaussian* (SWAG)^87^, which we will use in a combination with Deep Ensembles^83^ leading to *Multi-SWAG* as proposed by Wilson et al.^88^. In SWAG one interprets the stochastic gradient descent (SGD) algorithm, used to optimise the neural network given a loss function, as approximate Bayesian inference. SWAG estimates the first and second moment of the running SGD iterates to construct a Gaussian distribution over the weights $p(\theta|{{{{{{{\mathcal{D}}}}}}}}) \sim {{{{{{{{\mathcal{N}}}}}}}}}_{\bar{\theta },{{\Sigma }}}(\theta )$. Maddox et al. show that this Gaussian approximation suffices to capture the local shape of the loss space around the obtained minimum. When training a pre-trained neural network for *T* SWAG updates, the mean value and sample covariance are given as^87^

12 $$\bar{\theta }=\frac{1}{T}\mathop{\sum }\limits_{i=1}^{T}{\theta }_{i}$$

13 $${{\Sigma }}=\frac{1}{T-1}\mathop{\sum }\limits_{i=1}^{T}({\theta }_{i}-\bar{\theta }){({\theta }_{i}-\bar{\theta })}^{T}.$$

As computing the full covariance matrix is often intractable, SWAG approximates by splitting it into a diagonal covariance Σ_diag_, only containing the diagonal variances, and low-rank covariance Σ_low-rank_, which approximates the full matrix by only using the last few update steps. The diagonal covariance is given as

14 $${{{\Sigma }}}_{{{{{{{{\rm{diag}}}}}}}}}={{{{{{{\rm{diag}}}}}}}}(\overline{{\theta }^{2}}-{\bar{\theta }}^{2}),$$

where $\overline{{\theta }^{2}}=\frac{1}{T}\mathop{\sum }\nolimits_{i=1}^{T}{\theta }_{i}^{2}$ and the squares in ${\theta }_{i}^{2},{\bar{\theta }}^{2}$ are applied element-wise. For the low-rank covariance we first approximate Σ using the running estimate ${\bar{\theta }}_{i}$ after *i* steps: ${{\Sigma }}\;\approx\, \frac{1}{T-1}\mathop{\sum }\nolimits_{i=1}^{T}({\theta }_{i}-{\bar{\theta }}_{i}){({\theta }_{i}-{\bar{\theta }}_{i})}^{T}=\frac{D{D}^{T}}{T-1}$, where *D* is the deviation matrix consisting of columns ${D}_{i}=({\theta }_{i}-{\bar{\theta }}_{i})$. Further we only use the last *K* columns of *D* in order to calculate the low rank covariance matrix. Defining $\hat{D}$ as the matrix comprised of columns *T* − *K* + 1, …, *T* of *D*, we obtain

15 $${{{\Sigma }}}_{{{{{{{{\rm{low-rank}}}}}}}}}=\frac{\hat{D}{\hat{D}}^{T}}{K-1}.$$

Thus one only needs to keep track of $\bar{\theta },\;\overline{{\theta }^{2}}$ and $\hat{D}$ and can sample the weights used in Eq. (10) from the Gaussian ${{{{{{{\mathcal{N}}}}}}}}(\bar{\theta },\frac{1}{2}({{{\Sigma }}}_{{{{{{{{\rm{diag}}}}}}}}}+{{{\Sigma }}}_{{{{{{{{\rm{low-rank}}}}}}}}}))$. The full SWAG procedure is shown in Algorithm 1.

##### Algorithm 1

SWAG^87^

*θ*_0_ pre-trained weights; *η* learning rate; *T* number of training steps; *c* moment update frequency; *K* maximum number of columns in deviation matrix $\hat{D}$; *M* number of Monte Carlo samples in Bayesian model averaging

**Train** SWAG

 $$\bar{\theta }\leftarrow {\theta }_{0},\overline{{\theta }^{2}}\leftarrow {\theta }_{0}^{2}$$               ⊳ initialise moments

 **for**  *i* ← 1  **to**  *T*  **do**

  $${\theta }_{i}\leftarrow {\theta }_{i-1}-\eta {\nabla }_{\theta }{{{{{{{\mathcal{L}}}}}}}}({\theta }_{i-1})$$              ⊳ SGD update

  **if**  *mod*(*i*, *c*) = 0  **then**

   *n* ← *i*/*c*

   $$\bar{\theta }\leftarrow \frac{n\bar{\theta }+{\theta }_{i}}{n+1}$$, $$\overline{{\theta }^{2}}\leftarrow \frac{n\overline{{\theta }^{2}}+{\theta }_{i}^{2}}{n+1}$$           ⊳ update moments

   **if**  number of columns$(\hat{D})=K$  **then**

    remove first column in $\hat{D}$

   append column $({\theta }_{i}-\bar{\theta })$ to $\hat{D}$     ⊳ deviation matrix

 **return**  $${\theta }_{{{{{{{{\rm{SWA}}}}}}}}}=\bar{\theta },{{{\Sigma }}}_{{{{{{{{\rm{diag}}}}}}}}}=\overline{{\theta }^{2}}-{\bar{\theta }}^{2},\;\hat{D}$$

**Test**  Bayesian Model Averaging

 **for**  *i* ← 1  **to**  *M*  **do**

  draw $${\tilde{\theta }}_{i} \sim {{{{{{{\mathcal{N}}}}}}}}\left({\theta }_{{{{{{{{\rm{SWA}}}}}}}}},\frac{1}{2}{{{\Sigma }}}_{{{{{{{{\rm{diag}}}}}}}}}+\frac{\hat{D}{\hat{D}}^{T}}{2(K-1)}\right)$$

  $$p(y|{{{{{{{\rm{Data}}}}}}}}) +=\frac{1}{M}p(y|{\tilde{\theta }}_{i})$$

 **return** *p*( *y*∣Data)

In *Multi-SWAG* one combines this SWAG algorithm with deep ensembles by training multiple SWAG models and taking an equal amount of samples from each^88^.

### Neural network architecture and training

**Fig. 9:**
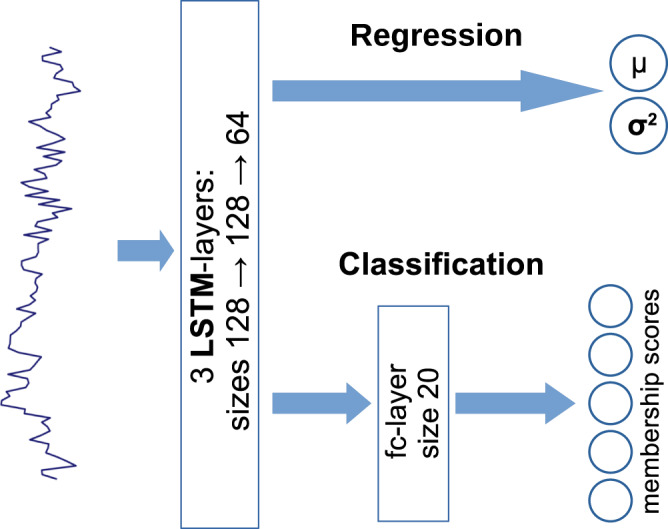
Architecture of the used Neural Network. For both regression and classification, the network first consists of three stacked long short-term memory (LSTM) layers^98^ of sizes 128, 128 and 64. For regression, the last LSTM is directly fully connected into the output layer returning a mean *μ* and variance *σ*, while for classification the output layer is preceded by another fully connected layer of size 20. The architecture is inspired by the successful applications of recurrent neural networks during the *AnDi-Challenge*^62,68,71,74^.

Inspired by its success in the *AnDi-Challenge*^62^ we chose a recurrent (LSTM^98^) neural network as depicted in Fig. 9 as our network architecture. We train separate networks for different trajectory lengths, but use the same architecture for each. Regardless of the trajectory length, all networks are trained on a total of 10^6^ trajectories from all 5 models. As stated above, for regression, the data set is equally distributed with respect to the anomalous diffusion exponents but not among ground truth models, and vice versa for classification. Later we also train networks on data sets consisting of only a single anomalous diffusion model and only 3 × 10^5^ trajectories. The neural network hyper-parameters, consisting of learning rate, weight decay^99^, batch size, training length (epoch number) and SWAG moment update frequency, are tuned using a separate validation set of 10^4^ trajectories, and final performance results are obtained from a third testing data set varying in size between 4 × 10^4^ and 1 × 10^5^, depending on the task. Data are generated using the andi-datasets python package^89^, shorter trajectories are obtained from the same data set by discarding later data points. Noise, as specified in Eq. (4), is added after cutting off the data points beyond the desired length, as otherwise the signal to noise ratio (snr) on the long trajectories may not represent the snr of the shortened trajectories, especially when dealing with models using a changing diffusivity like SBM.

Before training, the trajectory data sets consisting of time series of positions *x*_*t*_ are pre-processed by conversion to increments Δ*x*_*t*_ = *x*_*t*+1_ − *x*_*t*_ and normalising these increments to a standard deviation of unity for each trajectory. Rescaling the data in this manner speeds up the training process and, since we are not interested in a prediction of the diffusion coefficient, which would be altered by this step, it will not hinder the neural network’s performance.

The networks are trained using the *Adam* optimiser^100^ for 65 to 85 epochs with the last 10 to 15 epochs used for SWAG training, where one epoch corresponds to one full iteration through the training set. The exact epoch number as well as the other hyper-parameters are fine-tuned individually for each task and trajectory length using the validation data set. Once an optimal set of hyper-parameters is found, we use them to train 20 SWAG models and choose the 5 best-performing networks for *Multi-SWAG*, as measured by their achieved loss on the validation set. (This choice is necessary as some training processes may get trapped in suboptimal minima.) To obtain the final output, we sample 10 networks from each SWAG model for a total of 50 Monte Carlo samples and combine these into a single output of model probabilities for classification or mean and variance for regression in accordance to Eq. (10).

### Supplementary information

Supplementary Information

## Data Availability

The data resulting from applying the model on the test data sets are available at https://github.com/hseckler/BDL-for-AnDi. The training and test data sets were randomly generated using the andi-datasets python package^89^.

## Code availability

All software used in this study is available at https://github.com/hseckler/BDL-for-AnDi.
