# Author Correction: Bayesian deep learning for error estimation in the analysis of anomalous diffusion

**DOI:** 10.1038/s41467-023-43850-7

**Published:** 2023-11-30

**Authors:** Henrik Seckler, Ralf Metzler

**Affiliations:** 1 Institute for Physics & Astronomy, University of Potsdam, 14476 Potsdam-Golm, Germany; 2 Asia Pacific Centre for Theoretical Physics, Pohang, 37673 Republic of Korea

**Keywords:** Applied mathematics, Statistical physics

Correction to: *Nature Communications* 10.1038/s41467-022-34305-6, published online 07 November 2022

The original version of this Article omitted page numbers in references [1, 2, 4, 6, 7, 10, 11, 12, 13, 14, 19, 20, 21, 22, 23, 24, 25, 26, 27, 28, 29, 30, 31, 32, 33, 34, 35, 36, 37, 38, 42, 43, 44, 45, 47, 48, 50, 51, 52, 53, 54, 56, 57, 59, 60, 63, 66, 67, 68, 69, 70, 71, 72, 73, 74, 75, 76, 77, 79, 84, 88, 91, 94, 96, 98]. This has been corrected in the PDF and HTML versions of the Article.

Correct references are:

1. Pearson, K. The problem of the random walk. Nature 72, 294 (1905).

2. Okubo, A. Dynamical aspects of animal grouping: swarms, schools, flocks, and herds. Adv. Biophys. 22, 1-94 (1986).

4. Lüdtke, O., Roberts, B.W., Trautwein, U. & Nag, G. A random walk down university avenue: life paths, life events, and personality trait change at the transition to university life. J. Pers. Soc. Psychol. 101, 620 (2011).

6. Anderson, J. B. Quantum chemistry by random walk. H ^2^P, H^+^_3_ D_3*h*_^1^A′_1_, H_2_^3^Σ^+^_*u*_, H_4_^1^Σ^+^_*g*_, Be ^1^S. J. Chem. Phys. 65, 4121-4127 (1976).

7. Codling, E. A., Plank, M. J. & Benhamou, S. Random walk models in biology. J. R. Soc. Interface 5, 813-834 (2008).

10. Mises, R. V. Fundamentalsätze der Wahrscheinlichkeitsrechnung. Math. Z. 4, 1-97 (1919).

11. Einstein, A. Über die von der molekularkinetischen Theorie der Wärme geforderte Bewegung von in ruhenden Flüssigkeiten suspendierten Teilchen. Ann. Phys. 322, 549-560 (1905).

12. von Smoluchowski, M. Zur kinetischen Theorie der Brownschen Molekularbewegung und der Suspensionen. Ann. Phys. 326, 756-780 (1906).

13. Sutherland, W. A dynamical theory of diffusion for nonelectrolytes and the molecular mass of albumin. Philos. Mag. 9, 781-785 (1905).

14. Langevin, P. Sur la théorie du mouvement brownien. C. R. Acad. Sci. 146, 530-533 (1908).

19. Manzo, C. et al. Weak ergodicity breaking of receptor motion in living cells stemming from random diffusivity. Phys. Rev. X 5, 011021 (2015).

20. Krapf, D. et al. Spectral content of a single non-Brownian trajectory. Phys. Rev. X 9, 011019 (2019).

21. Stadler, L. & Weiss, M. Non-equilibrium forces drive the anomalous diffusion of telomeres in the nucleus of mammalian cells. New J. Phys. 19, 113048 (2017).

22. Kindermann, F. et al. Nonergodic diffusion of single atoms in a periodic potential. Nat. Phys. 13, 137-141 (2017).

23. Sokolov, I. M. Models of anomalous diffusion in crowded environments. Soft Matter 8, 9043-9052 (2012).

24. Bouchaud, J.-P. & Georges, A. Anomalous diffusion in disordered media: statistical mechanisms, models and physical applications. Phys. Rep. 195, 127-293 (1990).

25. Metzler, R. & Klafter, J. The random walk’s guide to anomalous diffusion: a fractional dynamics approach. Phys. Rep. 339, 1-77 (2000).

26. Saxton, M. J. Anomalous diffusion due to obstacles: a Monte Carlo study. Biophys. J. 66, 394-401 (1994).

27. Saxton, M. J. Anomalous subdiffusion in fluorescence photobleaching recovery: a Monte Carlo study. Biophys. J. 81, 2226-2240 (2001).

28. Burov, S., Jeon, J. H., Metzler, R. & Barkai, E. Single particle tracking in systems showing anomalous diffusion: the role of weak ergodicity breaking. Phys. Chem. Chem. Phys. 13, 1800-1812 (2011).

29. Ernst, D., Köhler, J. & Weiss, M. Probing the type of anomalous diffusion with single-particle tracking. Phys. Chem. Chem. Phys. 16, 7686-7691 (2014).

30. Höfling, F. & Franosch, T. Anomalous transport in the crowded world of biological cells. Rep. Prog. Phys. 76, 046602 (2013).

31. Horton, M. R., Höfling, F., Rädler, J. O. & Franosch, T. Development of anomalous diffusion among crowding proteins. Soft Matter 6, 2648-2656 (2010).

32. Tolić-Nørrelykke, I. M., Munteanu, E. L., Thon, G., Oddershede, L. & Berg-Sørensen, K. Anomalous diffusion in living yeast cells. Phys. Rev. Lett. 93, 078102 (2004).

33. Leijnse, N., Jeon, J. H., Loft, S., Metzler, R. & Oddershede, L. B. Diffusion inside living human cells. Eur. Phys. J. Spec. Top. 204, 377a (2012).

34. Metzler, R., Jeon, J. H., Cherstvy, A. G. & Barkai, E. Anomalous diffusion models and their properties: non-stationarity, nonergodicity, and ageing at the centenary of single particle tracking. Phys. Chem. Chem. Phys. 16, 24128-24164 (2014).

35. Montroll, E. W. & Weiss, G. H. Random walks on lattices. II. J. Math. Phys. 6, 167-181 (1965).

36. Hughes, B. D., Shlesinger, M. F. & Montroll, E. W. Random walks with self-similar clusters. Proc. Natl Acad. Sci. USA 78, 3287-3291 (1981).

37. Weissman, H., Weiss, G. H. & Havlin, S. Transport properties of the continuous-time random walk with a long-tailed waiting-time density. J. Stat. Phys. 57, 301-317 (1989).

38. Mandelbrot, B. B. & van Ness, J. W. Fractional Brownian motions, fractional noises and applications. SIAM Rev. 10, 422-437 (1968).

42. Zaburdaev, V., Denisov, S. & Klafter, J. Lévy walks. Rev. Mod. Phys. 87, 483 (2015).

43. Lim, S. C. & Muniandy, S. V. Self-similar Gaussian processes for modeling anomalous diffusion. Phys. Rev. E 66, 021114 (2002).

44. Jeon, J.-H., Chechkin, A. V. & Metzler, R. Scaled Brownian motion: a paradoxical process with a time dependent diffusivity for the description of anomalous diffusion. Phys. Chem. Chem. Phys. 16, 15811-15817 (2014).

45. Massignan, P. et al. Nonergodic subdiffusion from Brownian motion in an inhomogeneous medium. Phys. Rev. Lett. 112, 150603 (2014).

47. Cherstvy, A. G., Thapa, S.,Wagner, C. E. & Metzler, R. Non-Gaussian, non-ergodic, and non-Fickian diffusion of tracers in mucin hydrogels. Soft Matter 15, 2526-2551 (2019).

48. Makarava, N., Benmehdi, S. & Holschneider, M. Bayesian estimation of self-similarity exponent. Phys. Rev. E 84, 021109 (2011).

50. Bartumeus, F., da Luz, M. G. E., Viswanathan, G. M. & Catalan, J. Animal search strategies: a quantitative random-walk analysis. Ecology 86, 3078-3087 (2005).

51. Plerou, V., Gopikrishnan, P., Amaral, L. A. N., Gabaix, X. & Stanley, H. E. Economic fluctuations and anomalous diffusion. Phys. Rev. E 62, R3023 (2000).

52. Metzler, R. et al. Analysis of single particle trajectories: from normal to anomalous diffusion. Acta Phys. Pol. B 40, 1315-1330 (2009).

53. Magdziarz, M., Weron, A., Burnecki, K. & Klafter, J. Fractional Brownian motion versus the continuous-time random walk: A simple test for subdiffusive dynamics. Phys. Rev. Lett. 103, 180602 (2009).

54. Metzler, R. Brownian motion and beyond: first-passage, power spectrum, non-Gaussianity, and anomalous diffusion. J. Stat. Mech. 2019, 114003 (2019).

56. Condamin, S., Bénichou, O., Tejedor, V., Voituriez, R. & Klafter, J. First-passage times in complex scale-invariant media. Nature 450, 77-80 (2007).

57. Slezak, J., Metzler, R. & Magdziarz, M. Codifference can detect ergodicity breaking and non-Gaussianity. New J. Phys. 21, 053008 (2019).

59. Granik, N. et al. Single-Particle diffusion characterization by deep learning. Biophys. J. 117, 185-192 (2019).

60. Pinholt, H. D., Bohr, S. S. R., Iversen, J. F., Boomsma, W. & Hatzakis, N. S. Single-particle diffusional fingerprinting: A machine-learning framework for quantitative analysis of heterogeneous diffusion. Proc. Natl Acad. Sci. USA 118, e2104624118 (2021).

63. Aghion, E., Meyer, P. G., Adlakha, V., Kantz, H. & Bassler, K. E. Moses, Noah and Joseph effects in Lévy walks. New J. Phys. 23, 023002 (2021).

66. Park, S., Thapa, S., Kim, Y., Lomholt, M. A. & Jeon, J.-H. Bayesian inference of Lévy walks via hidden Markov models. J. Phys. A 54, 484001 (2021).

67. Thapa, S. et al. Bayesian inference of scaled versus fractional Brownian motion. J. Phys. A 55, 194003 (2022).

68. Argun, A., Volpe, G. & Bo, S. Classification, inference and segmentation of anomalous diffusion with recurrent neural networks. J. Phys. A 54, 294003 (2021).

69. Bo, S., Schmidt, F., Eichhorn, R. & Volpe, G. Measurement of anomalous diffusion using recurrent neural networks. Phys. Rev. E, 100, 010102 (2019).

70. Gentili, A. & Volpe, G. Characterization of anomalous diffusion classical statistics powered by deep learning (CONDOR). J. Phys. A 54, 314003 (2021).

71. Li, D., Yao, Q. & Huang, Z. WaveNet-based deep neural networks for the characterization of anomalous diffusion (WADNet). J. Phys. A 54, 404003 (2021).

72. Verdier, H. et al. Learning physical properties of anomalous random walks using graph neural networks. J. Phys. A 54, 234001 (2021).

73. Manzo, C. Extreme learning machine for the characterization of anomalous diffusion from single trajectories (AnDi-ELM). J. Phys. A 54, 334002 (2021).

74. Garibo-i-Orts, Ò., Baeza-Bosca, A., Garcia-March, M. A. & Conejero, J. A. Efficient recurrent neural network methods for anomalously diffusing single particle short and noisy trajectories. J. Phys. A 54, 504002 (2021).

75. Janczura, J., Kowalek, P., Loch-Olszewska, H., Szwabiñski, J. & Weron, A. Classification of particle trajectories in living cells: machine learning versus statistical testing hypothesis for fractional anomalous diffusion. Phys. Rev. E 102, 032402 (2020).

76. Kowalek, P., Loch-Olszewska, H., Łaszczuk, Ł., Opała, J. & Szwabiński, J. Boosting the performance of anomalous diffusion classifiers with the proper choice of features. J. Phys. A 55, 244005 (2022).

77. Loch-Olszewska, H. & Szwabiński, J. Impact of feature choice on machine learning classification of fractional anomalous diffusion. Entropy 22, 1436 (2020).

79. DeGroot, M. H. & Fienberg, S. E. The comparison and evaluation of forecasters. Statistician 32, 12-22 (1983).

84. MacKay, D. J. C. A practical Bayesian framework for backpropagation networks. Neural Comput. 4, 448-472 (1992).

88. Wilson, A. G. & Izmailov, P. Bayesian deep learning and a probabilistic perspective of generalization. Adv. Neural Inf. Process. Syst. 33, 4697-4708 (2020).

91. Kiureghian, A. & Ditlevsen, O. Aleatory or epistemic? Does it matter? Struct. Saf. 31, 105-112 (2009).

94. Wang, Q., Ma, Y., Zhao, K. & Tian, Y. A comprehensive survey of loss functions in machine learning. Ann. Data Sci. 9, 1-26 (2022).

96. Metropolis, N. & Ulam, S. The Monte Carlo method. J. Am. Stat. Assoc. 44, 335-341 (1949).

98. Hochreiter, S. & Schmidhuber, J. Long short-termmemory. Neural Comput. 9, 1735-1780 (1997).

