# Supplementary material for: Bayesian deep learning for error estimation in the analysis of anomalous diffusion
Source: Nat Commun. 2022 Nov 7;13:6717. doi: 10.1038/s41467-022-34305-6 (PMC9640593; doi:10.1038/s41467-022-34305-6)
Supplement: Supplementary file 1 — Supplementary Information [file 41467_2022_34305_MOESM1_ESM.pdf]

Supplementary information: Bayesian deep learning for error estimation in the analysis of anomalous diffusion

Henrik Seckler[1] and Ralf Metzler[1, 2]

[1] Institute for Physics & Astronomy, University of Potsdam, 14476 Potsdam-Golm, Germany
[2] Asia Pacific Centre for Theoretical Physics, Pohang 37673, Republic of Korea

S1. SUPPLEMENTARY METHOD 1

For the regression of the anomalous diffusion exponent α we evaluate the mean absolute error (MAE), defined as

$$\mathrm{MAE} = \frac{1}{N}\sum_{i=1}^{N} |\alpha_{i,\mathrm{gt}} - \alpha_{i,\mathrm{pred}}|, \quad (1)$$

where $\alpha_{i,\mathrm{gt}}$ and $\alpha_{i,\mathrm{pred}}$ are the ground truth and predicted anomalous diffusion exponent of the ith of the N trajectories contained in the test data set.

To quantify classification performance we use the accuracy, which is the fraction of correct classifications.

Reliability diagram and calibration error In order to assess the quality of uncertainty predictions we use reliability diagrams [1]. In these one illustrates observed errors as a function of predicted uncertainties. For classification tasks we divide the interval $[0, 1]$ into M bins $I_m = (\frac{m-1}{M}, \frac{m}{M}]$. If B_m is the set of trajectories with predicted confidences $p_i \in I_m$, then the accuracy in this interval is given as

$$\mathrm{acc}(B_m) = \frac{1}{|B_m|}\sum_{i\in B_m} \mathbf{1}(\hat{y}_i = y_i), \quad (2)$$

where $\hat{y}_i$,y_i are the ground truth and predicted model of input i. The mean predicted confidence of this set is

$$\mathrm{conf}(B_m) = \frac{1}{|B_m|}\sum_{i\in B_m} p_i, \quad (3)$$

where p_i is the predicted confidence of the model prediction of the ith input. In a perfectly calibrated model accuracy and confidence coincide for all bins, which corresponds to the diagonal in the reliability diagram. Any deviations from the identity represent miscalibration, which we summarise using the *expected calibration error* (ECE), defined as [1, 2]

$$\mathrm{ECE} = \sum_{m=1}^{M} \frac{|B_m|}{N} \left|\mathrm{acc}(B_m) - \mathrm{conf}(B_m)\right|, \quad (4)$$

where N is the number of samples in the test set.

Similarly, we can construct a reliability diagram for the regression of the anomalous diffusion exponent [3]. Here the roles of the mean confidence and the accuracy are taken by the predicted root mean variance (RMV) and the observed root mean squared error (RMSE). By introducing a binning of the predicted standard deviation into intervals $I_m = ((m-1)\Delta_\sigma, m\Delta_\sigma]$ of size Δ_σ, we define B_m as the set of trajectories with a predicted standard deviation $\sigma_{i,\mathrm{pred}} \in I_m$ and obtain

$$\mathrm{RMSE}(B_m) = \sqrt{\frac{1}{|B_m|}\sum_{i\in B_m} (\alpha_{i,\mathrm{gt}} - \alpha_{i,\mathrm{pred}})^2} \quad (5)$$

$$\mathrm{RMV}(B_m) = \sqrt{\frac{1}{|B_m|}\sum_{i\in B_m} (\sigma_{i,\mathrm{pred}})^2}. \quad (6)$$

As above, coinciding RMSE and RMV in all bins represent a perfectly calibrated model. Deviations from the ideal error prediction are represented by the *expected calibration error* (ECE)

$$\mathrm{ECE} = \sum_{B_m} \frac{|B_m|}{N} |\mathrm{RMV}(B_m) - \mathrm{RMSE}(B_m)|, \quad (7)$$

which can be modified to obtain the *expected normalised calibration error* (ENCE) [3]

$$\mathrm{ENCE} = \sum_{B_m} \frac{|B_m|}{N} \frac{|\mathrm{RMV}(B_m) - \mathrm{RMSE}(B_m)|}{\mathrm{RMV}(B_m)}. \quad (8)$$

S2. SUPPLEMENTARY FIGURES

Here we provide additional figures with details referenced in the main text.

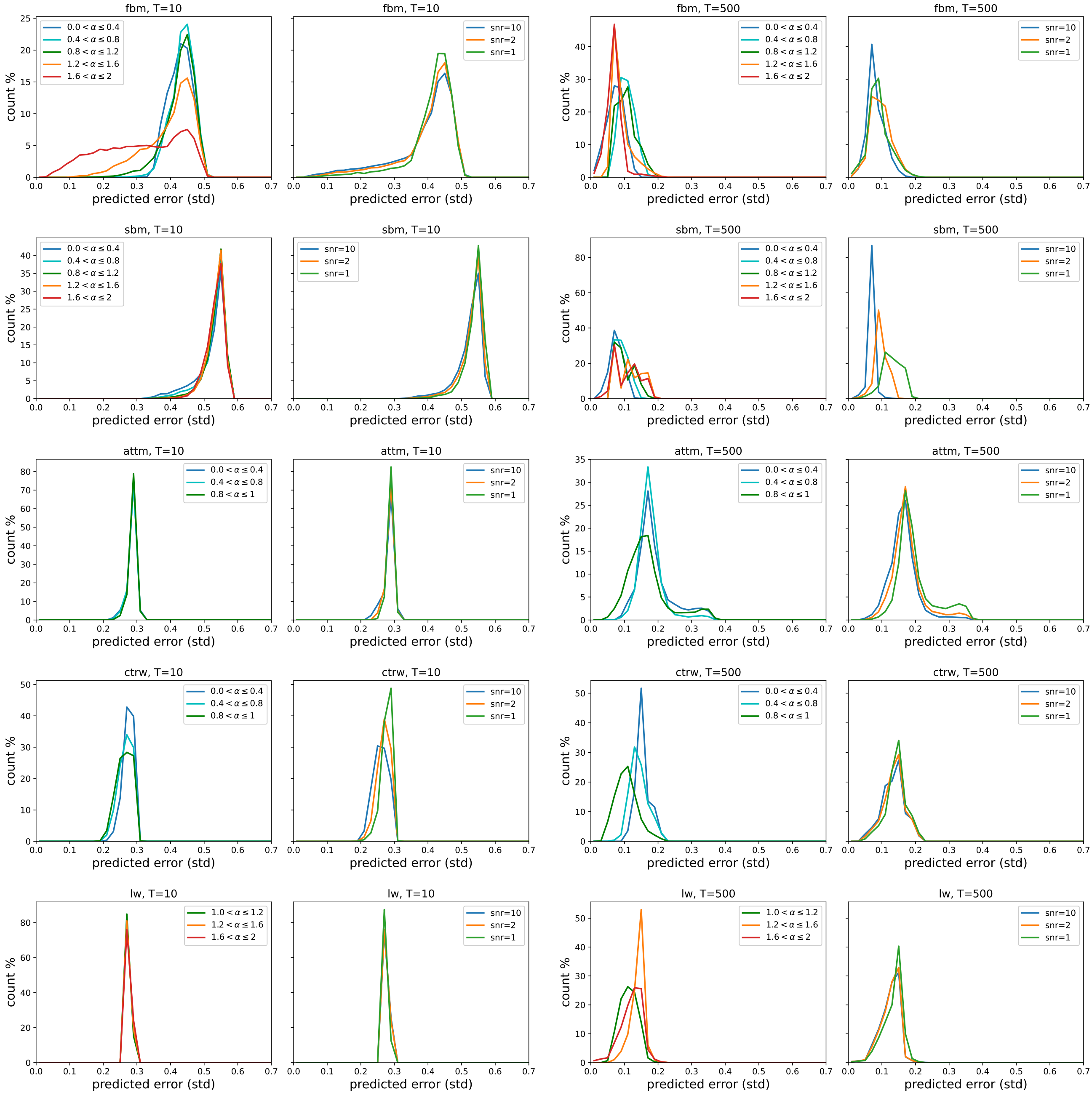

FIG. S1: Predicted error histograms split by exponent and noise for each model for lengths $T = 10$ and $T = 500$. The used networks were trained on data sets only containing the one respective diffusion model and the results are obtained from predictions based on 5×10^4 (FBM,SBM) or 4×10^4 (ATTM,LW,CTRW) trajectories.

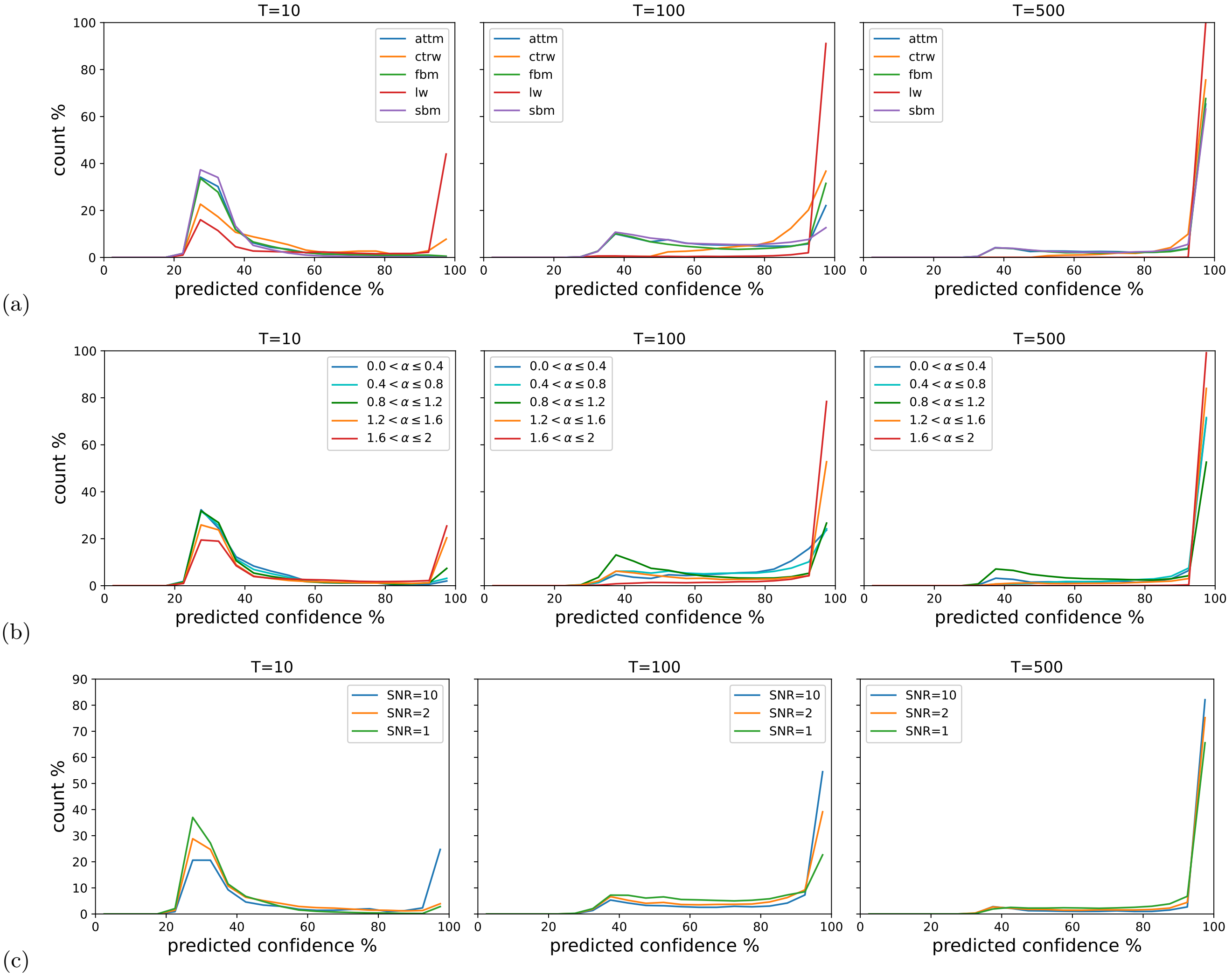

FIG. S2: Error histograms for classification split by (a) ground truth model, (b) ground truth exponent, and (c) by the used noise.

[1] C. Guo, G. Pleiss, Y. Sun, and K. Q. Weinberger, *On Calibration of Modern Neural Networks*, Int. Conf. Machine Learning, arXiv: 1706.04599 (2017).

[2] M. P. Naeini, G. Cooper, and M. Hauskrecht, *Obtaining well calibrated probabilities using Bayesian binning*, 29th AAAI Conf. Artif. Intell. (2015).

[3] D. Levi, L. Gispan, N. Giladi, and E. Fetaya, *Evaluating and calibrating uncertainty prediction in regression tasks*, arXiv:1905.11659 (2020).
